# Generative AI for climate governance and acceptability-constrained policy design

**DOI:** 10.1038/s44168-026-00362-6

**Published:** 2026-03-24

**Authors:** Ajaykumar Manivannan, Viktoria Spaiser, Tristan J. B. Cann, James Evans, Jordan P. Everall, Max Falkenberg, David Garcia, Weisi Guo, Rico Herzog, Ilona M. Otto, Yannick Oswald, Nicolò Pagan, Max Pellert, Charlie Pilgrim, Carlos Rodriguez-Pardo, Indira Sen, Alexander Sasha Vezhnevets

**Affiliations:** 1 School of Politics and International Studies, University of Leeds, Leeds, UK; 2 Centre for Climate Communication and Data Science, University of Exeter, Exeter, UK; 3 Department of Sociology, University of Chicago, Illinois, US; 4 Wegener Center for Climate & Global Change, University of Graz, Graz, Austria; 5 Department of Network and Data Science, Central European University, Vienna, Austria; 6 Department of Politics and Public Administration, University of Konstanz, Konstanz, Germany; 7 Centre for Assured & Connected Autonomy, Cranfield University, Cranfield, UK; 8 City Science Lab, HafenCity University Hamburg, Hamburg, Germany; 9 Institute of Geography and Sustainability, University of Lausanne, Lausanne, Switzerland; 10 Department of Informatics, University of Zurich, Zurich, Switzerland; 11 Barcelona Supercomputing Center, Catalonia, Spain; 12 School of Mathematics, University of Leeds, Leeds, UK; 13 Politecnico di Milano, Milan, Italy; 14 Euro-Mediterranean Center on Climate Change (CMCC), Milan, Italy; 15 RFF-CMCC European Institute on Economics and the Environment (EIEE), Milan, Italy; 16 chair for Data-Science in the Economic and Social Sciences, University of Mannheim, Mannheim, Germany; 17 Google DeepMind, London, UK

**Keywords:** Science, technology and society, Social sciences

## Abstract

Climate policies often fail when they clash with cultural values, social identities, and fairness perceptions. We propose Acceptability-Constrained Climate Policy Design (ACCPD), using large language models as “cultural world models” to simulate public responses before implementation. By embedding LLMs in generative agent-based models and physical system simulators, ACCPD aims to enable policymakers to co-optimize for climate-policy efficacy and social legitimacy. We discuss methodological limitations regarding representation and LLM opacity.

## Introduction

The climate crisis demands urgent action, yet technically sound policies repeatedly fail upon contact with social reality^1^. France’s 2018 carbon tax increase sparked the Yellow Vest protests and was swiftly abandoned^2^. Wind farms face fierce local opposition despite clear climate benefits and local energy price reductions^3^. In Canada, polarization around carbon pricing led Mark Carney, ahead of the 2025 election, to drop the Liberal Party’s commitment to the tax, calling it “divisive” (CBC News, https://www.cbc.ca/news/politics/mark-carney-drops-carbon-tax-1.7484290). Urban congestion pricing schemes stall over fairness concerns^4^. Across these cases, a common thread emerges: policies optimized for emissions reduction falter when they overlook how communities assess risk, fairness, and identity implications (i.e., whether a policy affirms or threatens their cultural values and social status). This can be exacerbated by opposition groups that will exploit these reservations to generate public backlash to these measures^5^. Public acceptance is thus critical for climate policies to succeed^1,6,7^.

Researchers across disciplines (e.g., social^6^, psychology^8^, economics^9^, politics^10^) have tried to understand the determinants of climate policy acceptance among the public. Bergquist et al.^1^ found perceived fairness and effectiveness among the public as the chief determinants (based on 89 data sets from 33 countries). Others have attributed social, psychological, and political identities to play important roles^6^. Furthermore, climate policy acceptance can also be affected by disinformation and obstruction campaigns^5^. Societal responses do not occur in a vacuum: entrenched power structures (e.g., political actors and economic powers) often fight to maintain the status quo^11^. These underlying power dynamics are both embedded within cultural narratives and actively shape them, as interest groups and incumbent actors work to steer public discourse and resist transformative change^5^.

Understanding societal responses to climate policy is, in any case, imperative. Engaging with the cultural narratives, social identities, and norms through which people interpret climate change and climate politics is vital for inspiring climate action^12^. Governments already use a range of participatory and empirical methods to anticipate societal responses to climate policies. For example, in Stockholm, a seven-month congestion charge pilot combined with surveys and a referendum revealed tangible travel and air-quality benefits, shifting public opinion and enabling permanent adoption in 2007^13^. The United Kingdom’s House of Commons commissioned Climate Assembly UK (2020), a UK-wide citizen assembly on climate change, to convene a representative sample of the population to deliberate on net-zero pathways^14^.

While these participatory and empirical approaches have proven effective, they are often slow, resource-intensive, and limited in scope^15–17^. Pilots and assemblies require months of preparation; surveys capture snapshots rather than evolving dynamics; and most methods are difficult to couple directly to energy, transport, or climate system models. As a result, governments often lack tools to rapidly explore how different narratives, framings, or design choices might shape public responses at scale, especially under polarized and fast-moving information environments. Emerging Artificial Intelligence (AI) methods offer a way to complement, not replace, these established approaches.

Large language models (LLMs), trained on vast corpora of digitized discourse, embody a large fraction of cultural narratives on many issues^18,19^, including climate change and climate politics. Early studies show that LLMs can approximate survey responses of various social groups^20^ and craft persuasive messages with human-level (and sometimes greater) effectiveness^21,22^. These models can be combined with agent-based models (ABMs) to study collective adoption dynamics and political outcomes. However, they also highlight significant risks: bias toward digital data, overfitting to dominant narratives, lack of physical grounding (since risk is embodied)^20^.

In summary, previous works have provided insights into factors that influence climate policy acceptance among the public, and current participatory and empirical methods face practical limitations. To accelerate climate action, we require frameworks that take advantage of the scale and speed that modern computing and data can offer, and forecast the landscape of climate policy acceptance. The primary objective of such an approach is explorative design: utilizing computational methods to pre-screen thousands of policy permutations, project friction points, and explain resistance dynamics to support human decision-making.

We propose Acceptability-Constrained Climate Policy Design (ACCPD), a simulation framework that treats societal acceptability as a design constraint from the start. ACCPD uses LLMs as cultural world models—useful, if imperfect—then couples them to generative ABMs (GABMs) and physical world simulators (climate, hazards, infrastructure) to quantify a policy’s Acceptability Frontier (how socially feasible it is) and detect narrative tipping risks (where communication backfires). Acceptability^8^ here refers not to manipulation, but to designing socially legitimate policies that people can trust, support, and sustain. Crucially, this is not a technocratic substitute for participation, but an upstream tool to inform, target, and support it. It can also prevent policymakers from being misguided by misconceptions regarding public support for climate policy^23^.

This paper reviews ongoing applications of LLMs as cultural reservoirs, culturally-calibrated agents, or policy intervention tools and identifies existing methodological and application gaps. We then propose the conceptual architecture for the ACCPD framework, specify its theoretical components, illustrate potential applications, and conclude by discussing the limitations of this approach. Our primary goal is to articulate a ‘grand vision’ for a new paradigm in climate policy planning rather than to dictate a rigid technical specification. We therefore prioritize architectural flexibility in the main text to avoid ‘overdesigning’ the system at this nascent stage, while providing an implementation protocol in the Supplementary Information file.

## Current Landscape: Promise and Pitfalls

The following section outlines the promise and pitfalls of current LLM and ABM applications to social simulations, and identifies the critical gaps that our proposed framework seeks to address.

### LLMs as Synthetic Publics

Large language models can serve as synthetic publics, stand-ins for groups of people, to explore policy ideas quickly and cheaply. This connects to emerging concepts of “augmented democracy" where AI assists in public deliberation and representation^24,25^.

Argyle et al.^20^ demonstrated that LLMs prompted with “personas” can mimic average survey answers for U.S. subpopulations, introducing “algorithmic fidelity” to judge how close these models are to real people. Strachan et al.^26^ showed that responses given by LLMs can resemble the answers that humans (1907 participants) give when they use “theory of mind” reasoning (i.e., thinking about other people’s beliefs, intentions, knowledge, ignorance), i.e., matching outputs of mentalistic inferences (but not the process itself). They recommend systematic testing for non-superficial comparison and deviations between LLM and human responses^26^. Lee, Sanguk, et al.^27^ showed in simulating survey responses that (2310 participants), with appropriate conditioning (demographic and specific covariates such as interpersonal discussion on the topic), LLMs can better predict attitudes and beliefs (53% to 91%) in global warming (along with voting behavior) than unconditioned models. Similarly, Qu, Y., & Wang, J.^28^, showed that LLMs can effectively simulate survey responses (400,000 participants from 100 countries), but their efficacy is limited to United States (refer to^29^ for similar results), and recommends diverse data sets (to train LLMs) for global applicability and to reduce bias for underrepresented demographics. In addition, Lutz et al.^30^ find that using demographic cues like names and interview-style prompts for role adoption improves the LLMs’ ability to represent different demographics. For a comprehensive review on the demographic representativeness of LLMs, we refer the reader to work done by Indira et al.^31^.

Thus, studies highlight that LLMs’ ability to simulate survey responses shows promise as synthetic publics that can imitate human personas, while showcasing best practices for improving accuracy. However, there are limitations: across different surveys and settings, synthetic responses can shrink real-world variation, reverse the direction of the relationship between key variables, and change with small prompt tweaks or model updates^28,32^.

### Agent-Based Modelling in Socio-Environmental Systems

If LLMs can approximate attitudes and write effective messages, a potential next step is linking them to simulations of collective and individual behavior.

Agent-based models (ABMs) are computational simulations where autonomous agents, representing individuals or groups, interact within a virtual environment according to specific behavioral rules. Unlike equation-based models that simulate aggregate system changes (top-down), ABMs simulate the system from the bottom-up: global phenomena (like polarization or market crashes) emerge from the local interactions of thousands of individual agents. This makes them uniquely suited for studying complex adaptive systems where social outcomes are non-linear.

Farmer and Foley^33^ argue that ABM is superior for capturing the heterogeneity and non-equilibrium dynamics inherent in complex adaptive systems, critical factors that traditional models, by relying on population averages and equilibrium, systematically overlook. This approach is particularly valuable in climate policy, where economic, social, and technological systems interact^34^. Recent work specifically addresses cultural change and climate-related policy through ABMs^35,36^.

A generative agent-based modelling framework (GABM), powered by LLMs as talking agents, thus provides an environment to simulate plausible discussions, augmenting the capabilities of traditional ABMs. For example, by holding realistic multi-agent conversations and forming shared norms in sandbox worlds, bridging language-level cultural models and ABMs^37,38^. Integrating LLMs with ABMs is especially promising to simulate collective behavior in opinion dynamics^39^, as shown by an early example using GPT-2 to enable agents to express verbally in opinion exchanges^40^. However, recent work also points to substantial challenges with LLM and ABM integration^41^.

In our pursuit of understanding societal responses to climate policy, we can take advantage of the discussed components (LLMs, ABMs) and shape them towards the exploration of climate policy acceptance, equipped with guardrails (validation, ethical, and governance frameworks).

## The ACCPD Framework: A Proposal for Co-Designing Impact and Legitimacy

**Fig. 1:**
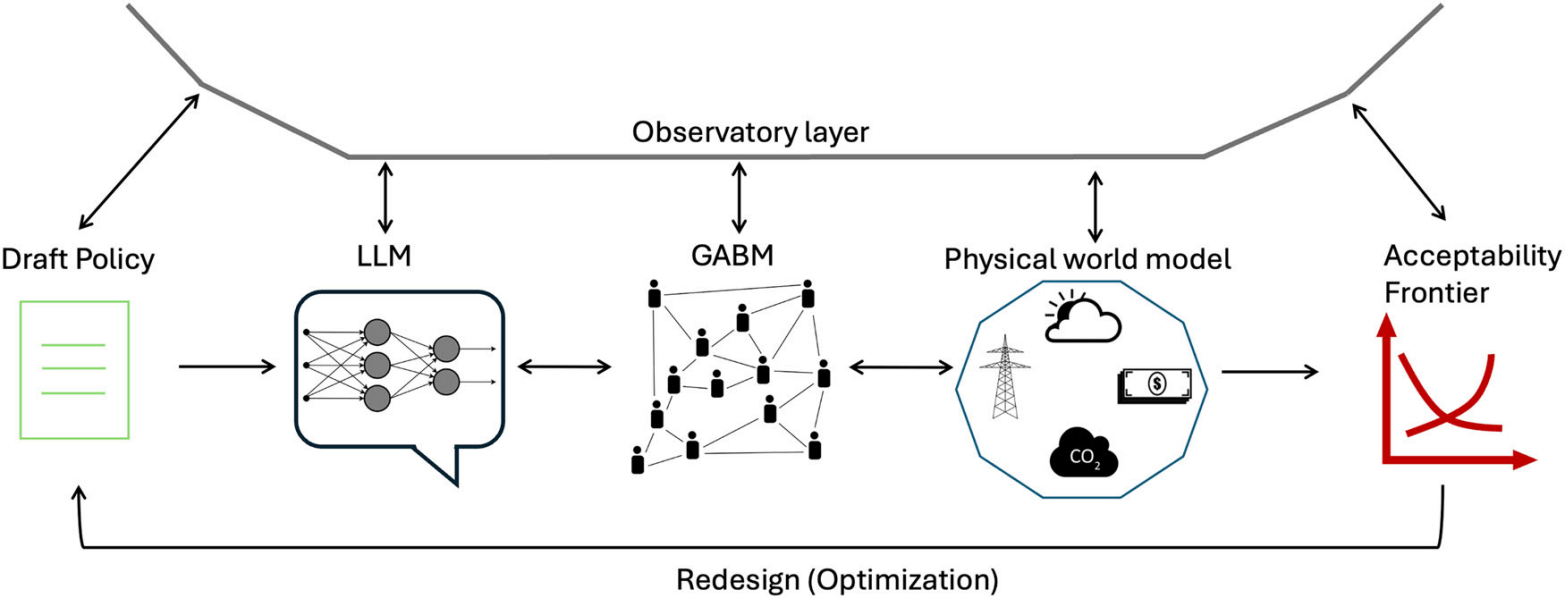
ACCPD framework (conceptual illustration): The diagram illustrates the iterative loop between policy design, social simulation, and physical constraints. The Observatory Layer (curved line) acts as an enveloping ‘Human-in-the-Loop’ interface where stakeholders define the Draft Policy and monitor the system. Inside the simulation, LLM-Agents assess the policy and diffuse opinions through a Social Network (GABM). These social outcomes interact with a Physical World Model (e.g., climate impacts), creating a feedback loop between social sentiment and physical reality. Finally, the Acceptability Frontier synthesizes these results to guide the Redesign (Optimization) step, where policymakers utilize the output to adjust policy parameters for higher political viability before re-assessment. We refer the readers to Supplementary Information Fig. S1 for the proposed comprehensive system architecture. This figure includes public domain icons from Wikimedia Commons.

We propose Acceptability-Constrained Climate Policy Design (ACCPD) as a framework for integrating socio-cultural dynamics into policy design from the outset. This approach would: (a) use LLMs as cultural world models to map narratives and fairness concerns, (b) couple these with social dynamics via GABMs, linked to physical world simulators, and (c) monitor the system through continuous validation using real-world signals (refer to Fig. 1). The aim is to locate and ethically expand the Acceptability Frontier, the set of designs achieving climate impact while remaining socially tenable^42,43^. Importantly, “acceptability” involves not just the general population but also institutions, businesses, media, and particularly marginalized communities. Hence, the term ‘societal acceptance’ or ‘societal response’ encompasses all these actors.

The proposed ACCPD framework consists of the following components: A. LLMs, B. GABMs, C. Physical world models, D. Acceptability frontier, and E. Observatory layer. The following defines each of their roles and potential in augmenting climate policy design.

### LLMs: Cultural World Modeling

Within the ACCPD framework, LLMs can be conceptualised as “cultural world models”, technological systems that can aggregate patterns from billions of lines of digitized human discourse^18^. Kozlowski et al.^19^ demonstrated this empirically: an LLM trained before COVID-19, when conditioned with political identities and exposed to pandemic facts, reproduced the partisan polarization that later emerged in reality. This suggests LLMs can “roll forward” societal processes within specified contexts.

When carefully prompted and calibrated, LLMs could potentially help anticipate how different publics might respond to policies. They can surface whose values are affirmed, what fairness concerns emerge, where identity sensitivities are triggered, and which counter-narratives gain salience. The goal is, however, not to substitute for human voices but to flag potential narrative risks and opportunities for subsequent testing with real-world data or for further in-depth citizen deliberation.

### GABMs: From Narratives to Social Cascades

GABMs comprise agents imbued with the capabilities of LLMs, allowing them to interact with each other and their environment through natural language rather than a set of predefined rules. As a result, they allow us to create virtual societies with programmable environments^38^. This, in turn, enables us to study the emergence of collective communication behavior under various contexts. In our ACCPD framework, GABMs serve to extend socio-cultural policy assessments to social dynamics of policy support or rejection patterns. These models would simulate how initial responses spread through social networks, accounting for: (a) network effects: opinion clustering and spreading along social ties^44^; (b) threshold dynamics: tipping points where fence-sitters follow early adopters^45^; (c) counter-mobilization: opposition organizing and spreading competing narratives^46^; and (d) complex contagion: how societal responses spread differently than simple information^47–49^. While ABMs already provide these capabilities, GABM provides a natural and richer form of communication and behavior, allowing us to represent more complex social situations^38^. In addition, we can also incorporate power dynamics through network characteristics to account for the role of power actors driving policy acceptance or rejection.

Existing GABM frameworks like Concordia^38,50^ already show that such infrastructure can be developed, providing a practical pathway for its implementation. Other GABM frameworks focus only on interaction on social media, like OASIS^51^, capturing verbal interaction within social and algorithmic systems.

We acknowledge that GABM is not a singular solution for all modeling challenges. Traditional mathematical approaches, such as Equation-Based Modelling (EBM) or System Dynamics^52^, remain superior for simulating aggregate flows or systems with well-defined physical laws, offering transparency and lower computational costs compared to the high-dimensional parameter space of LLM-based agents. However, these methods lack the capacity for semantic processing, the ability to interpret and generate natural language justifications based on distinct cultural identities.

### Physical world model

An integrated system that couples social simulation with physical world models, such as climate models, allows us to see the policy acceptance or rejection impacts on the physical world and the impact of a changing physical world on policy acceptance and rejection dynamics. While either can serve simply as inputs to the other (instead of a coupled system), this integration allows us to study policy adaptation patterns linked to measurable outcomes in the physical world (e.g. reduction of GHG emissions).

In simulating such a coupled framework, the societal response dynamics ultimately lead to either the acceptance or rejection of a policy. If a policy is accepted, it is implemented in the model, producing physical world implications such as reduced CO2 emissions. Conversely, if the policy is rejected, this too has physical world implications, such as continued GHG emissions or inadequate preparedness for extreme weather events. Integrated Assessment Models (IAMs) represent one possible class of models for this purpose, as they are designed to couple climate, energy, economic and land-use systems to project GHG emission pathways and evaluate mitigation and adaptation strategies^53,54^. However, traditional IAMs typically assume policy implementation as exogenous^55,56^, commonly overlooking the social dynamics that determine whether policies are adopted or abandoned—a gap that ACCPD explicitly addresses by making social acceptability endogenous and a key component of policy design.

Crucially, this layer is not limited to climate systems. It is designed as a modular interface to incorporate diverse domain-specific simulators, ranging from bottom-up physical system models (e.g., transportation, electricity grid) to economic models (e.g., energy pricing model), depending on the specific policy context. To demonstrate the framework’s capacity to integrate with established engineering tools across different scales, we reference two distinct implementation classes: micro-grid simulations for local energy policy can be done using GridLAB-D^57^) and national-scale climate risk and adaptation assessment to inform resilience planning can be performed by OpenCLIM^58^. However, developing suitable interfaces between social and physical models remains a significant challenge (e.g., time compatibility where one system operates in a different time scale than the other).

### The Acceptability Frontier

In the Acceptability Frontier (AF), the term “Acceptability" refers to the concept of social acceptance. While conventionally defined as meeting a minimum threshold of public support necessary for a policy to remain viable^59–61^, the ACCPD framework proposes to treat acceptability not as a fixed binary variable, but as a continuous objective.

As previously noted (see Introduction), the determinants of climate policy acceptance range from social legitimacy and fairness concerns^1^ to the influence of entrenched powerful actors (e.g., fossil fuel industry employing disinformation tactics to weaken public support for climate policies such as renewable energy deployment^5^). Consequently, the specific threshold for ‘viability’ is a political factor, set and adjusted by human stakeholders, often informed by decision-support frameworks such as Multi-Criteria Decision Analysis (MCDA)^62^ or PESTEL (Political, Economic, Social, Technological, Environmental, and Legal)^63^ analysis.

The proposed Acceptability Frontier itself is the technical component facilitating this decision. By treating social acceptance as one objective and other policy factors, such as CO_2_ emissions and implementation costs, as additional objectives, we can identify an optimal policy through multi-objective optimization. The boundary of these optimal trade-offs is what we call the “Acceptability Frontier.” This is akin to a Pareto Frontier, which identifies a set of equally optimal policy options where different compromises are possible (refer to Supplementary Information file for further details).

We note that the multiple objectives are not necessarily equivalent in the context of optimization. For instance, if the overall goal is to limit warming to well below 2 °C, atmospheric CO_2_ cannot exceed a specific threshold; this is a fixed constraint. This limits the flexibility of the emission reduction objective within a multi-objective optimization process. In contrast, acceptability is not fixed; it can be influenced and adjusted. Therefore, optimizing for acceptability necessarily involves an iterative process of adjusting a policy or its communication to increase support while maintaining emission reduction goals. The ACCPD framework can be used to test these potential adjustments.

The frontier can be dynamic and expanded through strategic choices. For example, if a proposed climate infrastructure is projected to face resistance within a community, resulting in a lack of acceptable choices, we can look at different measures that allow us to expand this acceptability frontier. This includes (a) benefit redistribution that ensures affected communities capture value^42^, (b) stakeholder engagement that ensures meaningful participations in decisions^64^, (c) phased implementation such as running initial pilot studies where positive benefit experience by the community drives support beyond early adopters^13^, and (d) narrative reframing, for instance emphasizing co-benefits like jobs^64^ or resilience or appealing to our moral obligation of preventing harm and protecting others^65^. For narrative reframing, LLMs’ persuasive capabilities can be leveraged. The ACCPD framework can be used to systematically test how different policy design choices shape public responses.

### The Observatory Layer: Monitoring, Validation, and Transparency

To keep ACCPD grounded and reproducible, we include an “observatory” layer that functions as a monitoring and audit module. Its role is to (i) compare model outputs with empirical signals (e.g., surveys, planning documentation, and other appropriate public indicators of response), (ii) maintain versioned records of datasets, prompts, parameters, and model configurations to enable reproducibility, and (iii) support structured recalibration when simulated trajectories diverge from observed signals.

The observatory can function as a hybrid-intelligence interface where communities, experts, policy makers, and other stakeholders examine results, question assumptions, and collectively adjust parameters. This layer does not prescribe a new governance institution; rather, it specifies a set of operational functions that can be carried out by existing oversight arrangements (e.g., research governance, regulatory review, ethics processes, or independent audits). In practice, stakeholders can (e.g., policy makers, civil service, city councils, citizen climate assemblies, etc.) act as the users of the system, utilizing the Acceptability Frontier to identify viable design constraints. Our focus on democratic application is deliberate, as recent literature demonstrates that participatory approaches are highly effective mechanisms for ensuring the long-term viability of climate infrastructure^52,66^. Therefore, our objective is to complement these proven participatory systems rather than deliver a framework that is agnostic to political governance. Yet, ACCPD may also find usage in other governance systems that may seek public buy-in for climate mitigation and adaptation projects^67^.

However, implementing such a system faces enormous challenges in data accessibility (e.g., tightening API policies of news and social media platforms), data integration, computational resources, and institutional coordination. It is worth noting that more data may not correspond to a more robust system. The choice of real-world signals should also depend on the required level of fidelity in the model’s key indicators, determined by the problem context.

## Northern Pass Transmission Line Project: An illustrative case study

This case study is presented as a retrospective hypothetical application to illustrate the logical workflow of the ACCPD framework. It demonstrates how the framework would be operationalized, rather than reporting novel simulation data.

The Northern Pass Transmission Line project (NPTL) in New Hampshire, United States (US), illustrates how technically optimized climate infrastructure can fail when social acceptability is overlooked. The project^64^, proposed in 2010, aimed to deliver low-carbon hydroelectric power from Quebec (Canada) to the New England area (US) through a 192-mile transmission corridor (1.6 billion US$ project set to deliver 1090 megawatts of electricity). The transmission was set to pass through Franconia Notch State Park and White Mountain National Forest, known for their scenic mountains and woods.

The project was estimated to have several benefits: (a) lowering electricity price estimated to be 150^68^ to 600 million $ per year^69^, (b) reduce 3.5 million tons of CO2 emissions per year^70^, (c) contribute $30 million per year to state and local taxes^68^, and $200 million grants to support conservation and development along the Northern Pass corridor (Citizens Count, https://www.citizenscount.org/issues/northern-pass), and (d) create 2600 jobs^69^.

Yet despite technical, climate-mitigation, and economic merits, the project faced fierce local opposition^64^. The downsides pointed out by critics included (a) altering the landscape of regions known for their natural beauty, (b) reducing tourism, and (c) reducing property values adjacent to the transmission lines. The project owners, after years of continued opposition, made several concessions, such as partially buried lines that do not impact the landscape view, alternate routes, and reduced capacity^64^. However, some of the concessions arrived too late, were insufficient in the view of the critics, and ignored communication with key stakeholders^64^. In addition, the project owners engaged in aggressive land buying, competing in purchases with opposition groups (e.g., pro-conservation organizations). Ultimately, the project was abandoned in 2019, after nearly a decade of regulatory and legal challenges (NHPR, https://www.nhpr.org/northern-pass.

It is argued that most of the opposition could have been avoided if the project had allowed for the transmission line to be buried entirely^64^, but it was refused by NPTL, citing costs (50% increase in cost according to NPTL project head, while others estimated it to be ≈ 25%^68^). There were clear signs from the very beginning that public opinion was polarized^64^. In fact, since the 1980s, many proposed energy infrastructures (transmission lines, nuclear plants, wind turbines) have failed or faced fierce opposition, for the same reasons echoed in the 2010s for the NPTL project (NH Magazine, https://www.nhmagazine.com/understanding-northern-pass/). In addition, the nearly nine-year struggle has allowed the opposition to grow and lock hands (NHPR, https://www.nhpr.org/northern-pass).

An ACCPD pre-analysis would have operationalized this through the pipeline defined in “The ACCPD Framework” section: (a) Layer I: Cultural World Model, (b) Layer II: Social Interaction layer (GABM), (c) Layer III: Physical World Model, and (d) Layer IV: Acceptability Frontier (refer to the Supplementary Information file for more detailed ACCPD architecture proposal).

Narrative initialization (Layer I): First, the Cultural World Model would ingest historical local media (2010-2011) to generate diverse agent personas. This would have surfaced specific framings (Layer I output): (a) local landowners and conservation groups framing the project as industrial intrusion into nature, (b) environmental organizations divided between emphasizing climate benefits and habitat protection, (c) Indigenous communities in Quebec raising long-standing concerns on consent and land appropriation, (d) urban residents viewing the project simply as clean energy infrastructure, detached from local realities.

Simulation of resistance (Layer II): These agents would then populate the GABM (Social Interaction layer). Baseline runs could have projected opposition emerging through local conservation networks, leading to protests, lawsuits, and a multi-year permitting stalemate, mirroring the project’s real-world fate (Layer II output).

Physical-social feedback (Layer III): The Physical World Model would then test alternate pathways. For instance, simulating the burial of transmission lines would generate new cost/visual parameters (Layer III input), which the agents would re-evaluate.

Optimization (Layer IV): Finally, the Acceptability Frontier would identify the Pareto-optimal design, revealing that while burying lines increases costs by 25%, it moves the project from the ‘Rejected’ zone into the ‘Acceptable’ zone, a trade-off the original developers failed to quantify until it was too late.

While some of these projections can be done through traditional surveys and consultations, the ACCPD framework could allow us to perform simulations in a fast-paced environment, taking advantage of LLMs’ ability to roll forward societal responses from the past (for example, the discourse since 1980s regarding similar infrastructures (NH Magazine, https://www.nhmagazine.com/understanding-northern-pass/), to project potential social patterns (in 2009 for NPTL project), when equipped with appropriate datasets (news articles, interviews, and council deliberations of the past) for specific contexts. The ACCPD proposes to cast policy design as an iterative search problem under explicit acceptability constraints. The key output is not one preferred policy, but a frontier of feasible designs that make the trade-offs between physical impact and social acceptability explicit, together with documented assumptions, subgroup heterogeneity, and uncertainty bounds. This allows decision-makers to explore alternatives systematically, identify where small design changes unlock large acceptability gains, and decide transparently which trade-offs are warranted in a given institutional context.

## Implementation Challenges and Requirements

### Methodological Limitations and Challenges

The proposed ACCPD framework currently faces substantial methodological hurdles. Yet, as highlighted in the “Current Landscape” section, recent studies suggest promising pathways to mitigate some of these challenges. The following lists the main limitations, challenges and possible solutions, several of which address more than one issue at the same time.

Representation gap: Even when LLMs match group averages, they can miss within-group diversity and minority voices^20,28,32^. Qu et al.^28^ show that LLMs underrepresent conservative, lower-income, less-educated, elderly, non-Western, non-English, and developing-nation populations. Crucially, these gaps are rarely accidental. They often reflect systemic power imbalances, a form of ‘digital colonialism’ where proprietary models generalize the values of their developers. Addressing this requires more than just better sampling; it requires building diverse datasets that cover multiple languages and communication forms, training region-specific models to reflect local contexts more accurately, encouraging the publication of data statements^71^, and working with communities through participatory design to include their own narratives and experiences. Open source LLMs like Apertus^72^ are increasingly developed with multi-lingual capabilities (1811 languages), open weights, and transparency to address ethical and diversity challenges.

Black Box Problem: LLM decision-making remains opaque. When an agent shifts from support to opposition, we cannot explain why, undermining trust and policy relevance. How will communities respond to policies shaped by inscrutable algorithms? Some solutions like explainable-AI methods^73^, open-weight and open-data architectures, and prompt-chain documentation can expose internal reasoning and enable public auditability.

Physical Grounding: While coupling social and physical models is standard in IAMs, ACCPD faces a unique semantic gap. Physical simulators output quantitative states (e.g., ‘Voltage drops by 5%’), but social agents react to qualitative narratives (e.g., ‘The government is neglecting our infrastructure’). The challenge lies in developing ‘Translation Middleware’ (Layer III) that converts engineering failures into accurate social signals without introducing hallucinations or narrative bias.

Hierarchical Validation & Quality Criteria: The validity of the ACCPD framework must be assessed at two distinct levels: component and system. At the component level, for example in the LLM layer, the primary quality criterion for the ‘Cultural World Model’ is Algorithmic Fidelity^20^, the statistical correlation between synthetic agent responses and empirical human data (e.g., Pew Research surveys). Before any simulation begins, agents must pass a ‘domain-specific Turing test’ where their baseline attitudes toward specific policy levers (e.g., carbon taxes) fall within the confidence intervals of the demographic groups they represent. At the system level, the framework should be validated using historical policy debates (such as the Northern Pass case) to verify if the model successfully identifies the specific friction points and outcome (e.g., rejection) that occurred in reality.

Purpose-built LLMs: Off-the-shelf models increasingly avoid sensitive political content by design. While important for safety, it can blunt research on real-world controversies. For research purposes, we may need less-restricted models that can represent contentious topics and actors. In addition, a locally trained LLM, say, one built on UK-specific language, media, and policy documents, can better reflect local norms, institutions, and edge cases.

### Practical Implementation Pathway

As a first step, near-term pilots could explore the potential of ACCPD in three phases.

In phase 1, minimal viable pilots need to be considered. Cities or utilities could test single policy levers (e.g., heat-pump subsidies): elicit attitudes using documented LLM prompts; validate with a 500-person survey; build a simple GABM with empirical social network data; couple to an existing sector model; publish a model card^74^ and validation plan; and compare projections to observed outcomes.

In phase 2, the interests of multiple stakeholders can be considered. Models, tested in phase 1, can be run for different scenarios to explore policy space (environmental justice communities, rural conservatives, urban progressives, business associations) and use the intersection of successful policies across groups for the final design.

In phase 3, infrastructure needed to support the ACCPD framework can be developed. For example, consider developing shared infrastructure such as GPUs and cloud computing to cut computational costs, bring multilingual training datasets to address diversity and representation concerns, standardize how the individual components and the overall framework of ACCPD can be validated, and design the governance framework for democratic and ethical application of ACCPD.

## Conclusion

Climate policies fail when they clash with cultural and social reality. ACCPD aims to offer a framework for making socio-cultural dynamics visible and manageable in policy design, treating acceptance as a core constraint from the start.

The potential is significant: rapid scanning of narrative landscapes, simulation of social cascades, and identification of policy configurations achieving both climate and social goals. Early pilots could demonstrate whether this approach can reduce policy failures and accelerate implementation.

ACCPD is best understood as a decision-support workflow: it can potentially help make assumptions explicit, map trade-offs, and identify where designs fail acceptability constraints; human judgment remains essential for setting objectives, interpreting uncertainty, and making legitimate choices. It aims to offer tools for exploring possibility spaces, or the Overton window (the range of policies acceptable to the mainstream population at a given time and in a given context), more quickly and broadly than traditional methods allow. But these proposed tools require careful development, continuous validation, transparent governance, and meaningful community control.

The climate crisis demands innovation in how we design and implement policy. ACCPD represents one attempt to bridge the gap between technical and environmental necessity (e.g., emissions targets and infrastructure requirements) and social possibility. Whether it fulfills this promise depends on our ability to address its limitations honestly, govern its use ethically, and ensure benefits flow to all communities, especially those historically excluded from both digital discourse and climate policy decisions.

Moving forward requires humility about what models can capture, vigilance about their misuse, and commitment to justice in their application. With these principles guiding development, ACCPD could contribute to more durable and equitable climate action. Without them, it risks becoming another tool that perpetuates existing inequities while failing to address the climate crisis.

## Supplementary information

Supplementary Information

## Data Availability

No datasets were generated or analysed during the current study.
