# Supplementary material for: Generative AI for climate governance and acceptability-constrained policy design
Source: NPJ Clim Action. 2026 Mar 24;5(1):37. doi: 10.1038/s44168-026-00362-6 (PMC13008763; doi:10.1038/s44168-026-00362-6)
Supplement: Supplementary file 1 — Supplementary Information [file 44168_2026_362_MOESM1_ESM.pdf]

Generative AI for Climate Governance and Acceptability-Constrained Policy Design

Ajaykumar Manivannan[1*], Viktoria Spaiser[1*], Tristan J.B. Cann[2], James Evans[3], Jordan P. Everall[4], Max Falkenberg[5], David Garcia[6], Weisi Guo[7], Rico Herzog[8], Ilona M. Otto[4], Yannick Oswald[9], Nicolò Pagan[10], Max Pellert[11], Charlie Pilgrim[12], Carlos Rodriguez-Pardo[13,14,15], Indira Sen[16], Alexander Sasha Vezhnevets[17]

[1*]School of Politics and International Studies, University of Leeds, Leeds, UK.
[2]Centre for Climate Communication and Data Science, University of Exeter, Exeter, UK.
[3]Department of Sociology, University of Chicago, Illinois, US.
[4]Wegener Center for Climate & Global Change, University of Graz, Graz, Austria.
[5]Department of Network and Data Science, Central European University, Vienna, Austria.
[6]Department of Politics and Public Administration, University of Konstanz, Konstanz, Germany.
[7]Centre for Assured & Connected Autonomy, Cranfield University, Cranfield, UK.
[8]City Science Lab, HafenCity University Hamburg, Hamburg, Germany.
[9]Institute of Geography and Sustainability, University of Lausanne, Lausanne, Switzerland.
[10]Department of Informatics, University of Zurich, Zurich, Switzerland.
[11] Barcelona Supercomputing Center, Catalonia, Spain.
[12]School of Mathematics, University of Leeds, Leeds, UK.
[13] Politecnico di Milano, Milan, Italy.
[14] Euro-Mediterranean Center on Climate Change (CMCC), Milan, Italy.
[15] RFF-CMCC European Institute on Economics and the Environment (EIEE), Milan, Italy.

[16]chair for Data-Science in the Economic and Social Sciences, University of Mannheim, Mannheim, Germany.
[17]Google DeepMind, London, UK.

*Corresponding author(s). E-mail(s): a.manivannan@leeds.ac.uk; V.Spaiser@leeds.ac.uk;
Contributing authors: t.j.b.cann@exeter.ac.uk; jevans@uchicago.edu; jordan.everall@uni-graz.at; max.falkenberg@protonmail.com; david.garcia@uni-konstanz.de; weisi.guo@cranfield.ac.uk; rico.herzog@hcu-hamburg.de; ilona.otto@uni-graz.at; y-oswald@web.de; nicolo.pagan@uzh.ch; max.pellert@bsc.es; C.P.Pilgrim@leeds.ac.uk; carlos.rodriguezpardo.jimenez@gmail.com; indira.sen@uni-mannheim.de; vezhnick@google.com;

1 Operationalizing ACCPD: A Logical Architecture and Implementation Protocol

Having established the conceptual foundations of the ACCPD framework, this section details its logical architecture and operationalization. This architecture is presented as a flexible protocol, serving as a template for practical implementation rather than a rigid standard.

We present an implementation protocol that integrates Large Language Models (LLMs) as Cultural World Models, Generative Agent-Based Models (GABMs), utilizing Google DeepMind's Concordia environment [1], and high-fidelity physical systems simulators. This architecture is designed to function as a computational sandbox wherein the collision of policy instruments, social narratives, and relevant systems (e.g., physical, economic) can be simulated, observed, and optimized. The objective is to locate the "Acceptability Frontier"—the Pareto-optimal set of policy configurations that maximize decarbonization efficacy without violating the threshold of social legitimacy required for durable implementation.

The following technical specification decomposes the ACCPD framework into five interoperable layers: (I) Cultural World Model for narrative and persona generation, (II) Social Interaction layer for simulating social dynamics, (III) Physical World Model layer to model relevant systems of interest, (IV) Acceptability Frontier layer to traverse the design space, and (V) Observatory layer to facilitate continuous validation and ethical governance. An overview of their topology is shown in Table S1 and Figure S1. It is worth noting that many of the examples mentioned below are derived from the Northern Pass Transmission Line case study described in Section 4 of the main article.

Table S1 ACCPD components overview

Layer	Component name	Function	Core Technology
Layer I	Cultural Model Model	Initialization: Static estimation of initial sentiment, narrative generation, and agent population synthesis.	Fine-tuned LLMs (e.g., GPT 5.2), RAG Pipelines [2], Vector Databases (e.g., Pinecone [3]).
Layer II	Social Interaction	Simulation: Dynamic modeling of opinion diffusion, polarization, and social cascades via generative agents.	Google DeepMind Concordia [1], NetworkX [4], Graph Theory.
Layer III	Physical World Model	Grounding: Simulation of biophysical impacts (emissions, costs, reliability) and infrastructure constraints.	e.g., GridLAB-D [5], IAM Integration, OpenCLIM [6].
Layer IV	Acceptability Frontier	Search: Automated traversal of the policy parameter space to identify the Acceptability Frontier.	Multi-Objective Evolutionary Algorithms (e.g., NSGA-II) [7], Pareto Optimization
Layer V	Observatory	Governance: Validation, real-time calibration, ethical guardrails, and bias mitigation.	Real-time Data Ingestion APIs, Bias Detection Engines, Model Cards.

1.1 Layer I Implementation: The Cultural World Model

The core of the proposed ACCPD framework would be the Synthetic Public, a population of digital agents designed to mirror the diversity of a specific human community. Layer I outlines how these agents could be generated to represent reality rather than generic stereotypes.

Constructing the Population (Data & Sampling) To build accurate agents, the proposed system would begin by ingesting real-world demographic data, such as Census records. If the target region were a specific county, the digital population would be generated to match that county's actual age, income, and education distribution.

Because demographics alone do not predict behavior, the proposal suggests enriching these profiles with psychological data from large-scale opinion surveys. For example, an agent representing a rural, conservative resident would be initialized with lower trust in federal institutions, reflecting empirical trends. To ensure minority voices are not drowned out, the system would deliberately oversample underrepresented groups, ensuring their perspectives remain mathematically significant in the simulation.

Adding Local Context: Standard AI models often act like "global average" citizens. To transform them into "local residents", the architecture proposes using Retrieval-Augmented Generation (RAG) [2]. This would involve building a knowledge base by indexing local newspapers, town hall transcripts, and planning documents.

When an agent is created, for instance, a "Hotel Owner in the White Mountains", the system would query this database for relevant local articles about tourism and

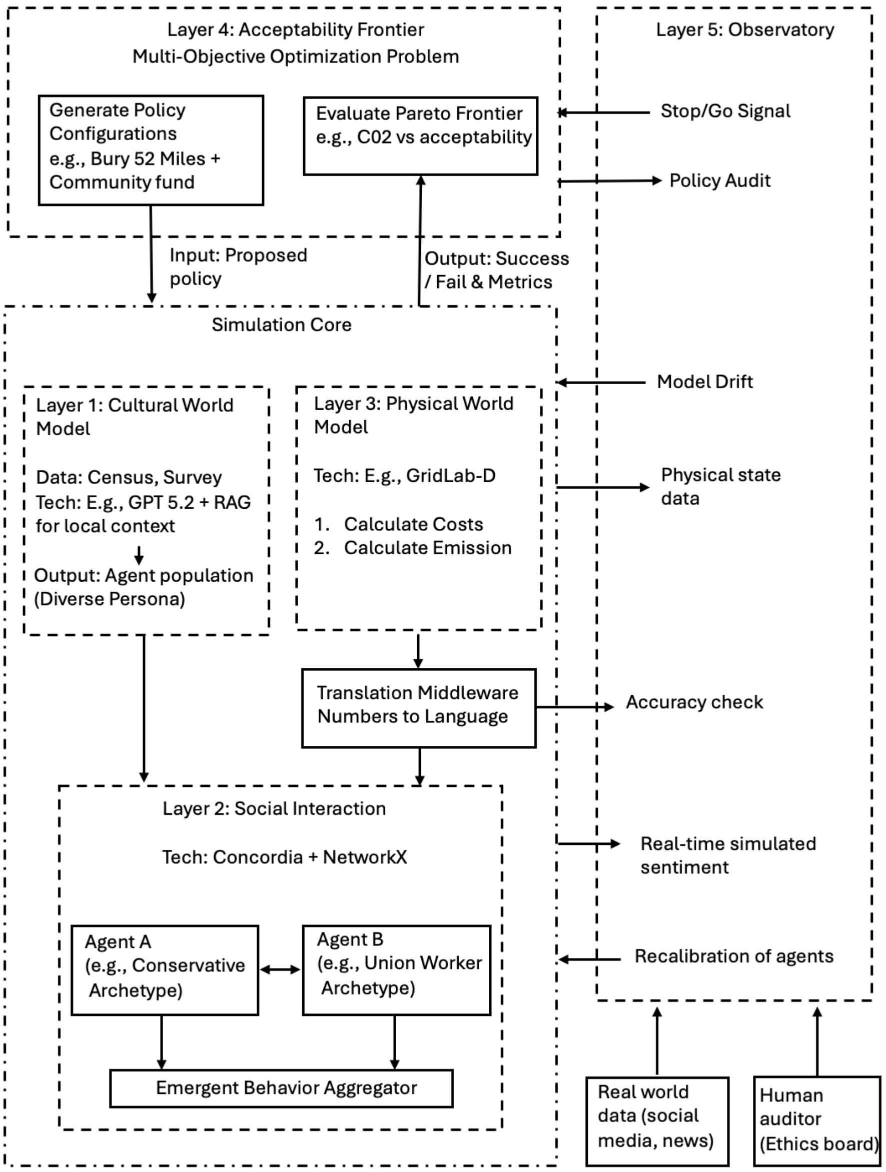

Fig. S1 ACCPD system flowchart

vantage points. This would allow the agent to debate using specific local vocabulary and economic anxieties, rather than generic complaints.

Mapping the Arguments: Before the simulation begins, the system can perform a static analysis to map the “Argument Space.” It would identify the core narratives held by different archetypes (e.g., conservationists vs. developers). This narrative dictionary would serve two purposes: giving agents specific topics to discuss, and

establishing a baseline so researchers could track when fringe ideas migrate into the mainstream discussion.

1.2 Layer II Proposal: Social Interaction (via Concordia)

To simulate how individual opinions evolve into collective action, the proposed framework adopts Google DeepMind's Concordia [1]. This library is purpose-built for Generative Agent-Based Modeling, allowing us to construct agents with rich psychology rather than simple rules.

Agent Architecture: Extending Concordia

In Concordia, an agent is defined by a stack of modular Components. To adapt this for political simulation, the proposal suggests extending the base library with three domain-specific modules:

- The PoliticalIdentity Component: This extends the standard Identity module. It stores the persona generated in Layer I (e.g., union worker, fiscal conservative) and acts as a cognitive filter. Using the Theory of Appropriateness [8], this component ensures agents evaluate policy not just on utility ("is this useful?"), but on identity congruence ("Is this what someone like me supports?").
- The TrustMatrix Component: To model polarization, this dynamic graph would sit in the agent's memory. It assigns weight to information sources. If an agent interacts with a like-minded peer, trust increases (homophily [9]). Conversely, if the State issues a statement that contradicts the agent's local observations (fed from Layer I), the trust score for the government degrades.
- The NarrativeInventory: Agents don't just speak, they also carry ideas. This component manages the specific arguments an agent currently holds. It can utilize a Virality Weight mechanism i.e., sensational rumors (e.g., "property value collapse") are weighted to spread faster than technical corrections.

The Game Master as the Environment

In Concordia [1], the Game Master (GM) is the entity that controls the clock, the location, and the global state. Here, the GM serves as the Media and Policy Interface.

Targeted Information Injection: The GM does not just broadcast news; it can simulate information asymmetry. It can deliver a "Jobs Report" component specifically to agents tagged as "Union Members", while sending "Conservation Warnings" only to the "Environmentalist" cluster. This heterogeneity drives the echo chambers often seen in real-world climate conflicts [10].

Social Network & Diffusion

Realistic diffusion requires a realistic network. The simulation can initialize agent connections using a Stochastic Block Model (a method for generating community clusters), using Network Science libraries like NetworkX (Python) [4].

- Clusters & Connectors: Agents can be tightly connected within their "Zip Code" and "Ideological Group."

- Bridge Nodes: The system explicitly identifies "Connectors", agents like a local journalist or school principal who link disparate groups. These nodes are critical: if a Connector adopts a negative narrative, they facilitate a "cascade," allowing opposition to spill from a fringe group into the mainstream.

The Simulation Loop (The Concordia Clock)

The simulation can run in discrete steps (e.g., 1 Step = 1 Day), following a rigorous cognitive cycle:

- Perception: Agents receive signals from the GM (News) and observations from their environment.
- Retrieval & Cognition: Using Concordia's Associative Memory [1], the agent retrieves relevant past experiences and filters the new signal through their PoliticalIdentity and TrustMatrix.
- Action Selection: The agent can then select a response ranging from "Passive Acceptance" to "Hard Resistance" (e.g., protesting).
- Interaction: Agents can converse with peers, potentially passing items from their NarrativeInventory to others.
- Reflection: At the end of the cycle, agents can synthesize their interactions into high-level summaries (e.g., "I realized today that the developers are ignoring our concerns"), effectively solidifying their stance for the next day.

1.3 Layer III Proposal: The Physical World Model

The proposed framework prioritizes physical grounding to anchor social dynamics in material reality. Social narratives do not exist in a vacuum; they are reactions to physical realities, whether that is the visual impact of a pylon, a sudden spike in electricity bills, or a basement flooding. Layer III is designed to simulate these realities and feed them back into the social layer.

The Bridge: Domain-Specific Languages (DSL)

Connecting a social simulation (people talking) to a physical simulation (complex engineering physics) is technically difficult. To implement this robustly, the framework relies on Domain-Specific Languages (DSLs) [11], a specific vocabulary that a computer program understands, to act as the intermediate layer between the social world and physical simulators. Combined with emerging modeling infrastructures in the digital twin space [12] and open standards, one can leverage DSLs to couple various models and scenario runs with the GABM layer.

As demonstrated by Dong et al. [11], Large Language Models (LLMs) can effectively bridge the gap between human intent and complex solvers by generating configuration scripts in a DSL. This allows the ACCPD system to be modular:

- Modularity: To swap "World Models" (e.g., changing from an electrical grid simulator to a flood model), developers simply replace the DSL vocabulary. The system then generates scripts for the new model without requiring a total code rewrite.

- Orchestration: While a standard LLM pipeline can handle this translation, the architecture can be designed to support a dedicated "Model Agent", an autonomous orchestrator, should the complexity of the simulation require dynamic management of multiple physical tools simultaneously.

Translating Numbers into News

Once the physical simulator executes the DSL script, it produces raw numbers (e.g., Rate increase = $0.03/kWh). The social agents, however, respond to stories, not spreadsheets. This step acts as a translation middleware.

The system can receive the raw data and use a specialized prompt to convert it into narrative signals tailored to different demographics:

- Input: The physical model calculates that burying power lines will raise costs.
- Translation for Low-Income Agents: "News Alert: Utility bills expected to rise by $40/month due to project changes."
- Translation for Environmentalist Agents: "Report: Burying transmission lines below ground forces a difficult trade-off with renewable energy affordability."

These narratives are injected into the simulation (Layer II), forcing agents to reconcile their aesthetic desires with their economic reality.

Syncing Time (The Feedback Loop)

Physical systems and social systems move at different speeds. A power grid changes in seconds; social opinions shift over months. To fix this, this layer proposes a Time-Bucketing approach:

- Aggregation (Social Step): The social model runs for a set period (e.g., one month). The system summarizes the collective result—for example, "Project permitting delayed by lawsuit."
- Simulation (Physical Step): The system passes this "Delay" variable to the physical model via the DSL. The model steps forward to calculate the consequences: accrued financial interest, increased carbon emissions, or reduced grid reliability.
- Feedback (Closing the Loop): These new physical realities are fed back into the social world. If the delay leads to a "reliability crisis" (a simulated blackout), the agents experience this event. This might drastically alter the narrative, shifting the conversation from "Stop the project" to "We need power, build it now!"

1.4 Layer IV Proposal: The Acceptability Frontier

The ultimate goal of the ACCPD framework is not merely to anticipate project failure, but to design for success. Layer IV proposes an optimization process to discover the Acceptability Frontier, the "sweet spot" where a project is both technically effective and socially supported.

A useful way to formalize this is through the Pareto Frontier (also called the Pareto front) [13, 14]. In ACCPD, each candidate policy configuration (a Policy Vector) can produce at least two outcomes after being evaluated through Layers I–III: a physical

outcome (e.g., emissions reduction) and a social outcome (e.g., support, compliance, protest risk). Because improving one outcome often worsens the other, we generally cannot identify a single globally "best" policy. Instead, what we want is the set of policies that are efficient in the trade-off sense.

A policy A is said to Pareto-dominate policy B if A is at least as good as B on all objectives, and strictly better on at least one. The Pareto Frontier is the set of non-dominated policies, those for which no other candidate simultaneously achieves higher physical impact and higher acceptability. In other words, the Pareto front is the boundary of "you can't improve one objective without paying a price in the other."

In ACCPD, we can interpret a specific subset/region of this Pareto front as the Acceptability Frontier: the boundary of implementable policy designs where social acceptance is sufficient for the physical benefits to be realized. This is not just "high acceptability"; it is the threshold region where policies avoid unacceptable social dynamics (e.g., mobilization cascades, lawsuits, non-compliance, or legitimacy collapse) while still producing meaningful emissions reductions.

Conceptually, the Pareto Frontier answers: What are the best achievable trade-offs between impact and acceptability, given our design space and model? The Acceptability Frontier answers: Where is the boundary between policies that can plausibly pass (and therefore deliver impact) versus policies that trigger social failure modes—even if they are physically efficient?

This distinction matters because a policy can be Pareto-efficient yet still non-viable (e.g., very high CO2 reduction but consistently triggering resistance dynamics). Layer IV therefore uses Pareto reasoning to structure the search, and uses acceptability constraints (from the Layer I–III simulation outcomes) to identify which Pareto-efficient designs are actually "buildable."

Multi-Objective Evolutionary Algorithm (MOEA)

We can formulate the policy design challenge as a Multi-Objective Optimization Problem (MOOP).

- Objective Function 1 (f_1): Maximize Physical Impact (e.g., Δ CO2 Emissions).
- Objective Function 2 (f_2): Maximize Social Acceptability (e.g., % of Population Support).

Since these objectives are often conflicting (e.g., the most efficient route is often the most visually intrusive), there is no single best solution. We can employ multi-objective evolutionary algorithms such as the Non-dominated Sorting Genetic Algorithm II (NSGA-II) [7] to identify the Pareto front.

The Optimization Loop

- Initialization: The Kernel (AF layer) can generate a population of N distinct Policy Vectors. A Policy Vector contains the tunable parameters of the proposal (e.g., different routes, different compensation levels for homeowners).
- Simulation (Evaluation): Each Policy Vector is then fed into the ACCPD simulation stack (Layers I-III). The simulation can run rapidly (using accelerated time steps) to determine the terminal state: Does the policy pass? What is the final CO2 reduction?

- Selection: The Kernel can rank the policies. Policies that result in "Social Collapse" (riots/lawsuits) are penalized. Policies that yield low carbon benefits are penalized.
- Crossover and Mutation: The algorithm can "breed" the high-performing policies. It might combine the "Route B" from Policy X with the "Community Fund" from Policy Y. It can introduce random mutations (e.g., "Try burying the transmission line only in the National Park").
- Convergence: Over successive generations, the algorithm may converge on a set of optimized policies. These represent the Acceptability Frontier.

1.5 Layer V Proposal: The Observatory

To ensure the framework remains a reliable and ethical tool for decision-making, the proposed architecture includes a final layer: The Observatory. This layer serves as a "reality check" and a transparency center, ensuring the simulation stays grounded in facts rather than AI-generated errors, it also offers a human-in-the loop mechanism for governance and stakeholder input.

Real-Time Validation

A major risk in any simulation is that it becomes a "black box" that loses touch with the real world. The Observatory is designed to continuously compare the digital simulation against real-world data to ensure it remains accurate.

- Sentiment Monitoring: The system can connect to live news feeds and public data sources to monitor how people are actually reacting to similar infrastructure projects in real time.
- Gap Detection: If the "Synthetic Public" in the simulation starts behaving very differently from real-world human behavior, the Observatory triggers a calibration halt.
- Automatic Adjustment: Instead of letting the simulation continue with errors, the system would pause to adjust the agents' settings. This ensures the model doesn't "hallucinate" a fake consensus or become overly optimistic about how easy a project will be to implement.

Ethical Safeguards and Fact-Checking

Because AI models are powerful, it is vital to ensure they are used to find genuine common ground rather than to mislead. The Observatory can enforce strict Guardrails to keep the simulation honest.

- The Fact-Check Filter: The system can include an automated audit tool that reviews the project designs suggested in Layer IV. If the optimization engine suggests a strategy based on incorrect or impossible information, for example, claiming a power line will be invisible when the physical model shows it will clearly be seen, the system can block that suggestion. The framework can thus be strictly limited to finding authentic solutions, not hiding facts.
- Transparency Reports: Following ethical AI standards, the Observatory can automatically generate a "report card" for every simulation. These documents clearly

list: (a) where the demographic data came from, (b) the known limitations of the AI models used, and (c) any potential biases in the physical or social data.

Governance and Human Oversight

Ultimately, the framework is designed as a Decision Support Tool, not a decision-maker. The Observatory can provide a "dashboard" for human experts and stakeholders to review the data, challenge the simulation's assumptions, and ensure that the final project designs respect the rights and concerns of the local community.

References

[1] Vezhnevets, A.S., Agapiou, J.P., Aharon, A., Ziv, R., Matyas, J., Duéñez-Guzmán, E.A., Cunningham, W.A., Osindero, S., Karmon, D., Leibo, J.Z.: Generative agent-based modeling with actions grounded in physical, social, or digital space using concordia. arXiv preprint arXiv:2312.03664 (2023)

[2] Lewis, P., Perez, E., Piktus, A., Petroni, F., Karpukhin, V., Goyal, N., Küttler, H., Lewis, M., Yih, W.-t., Rocktäschel, T., *et al.*: Retrieval-augmented generation for knowledge-intensive nlp tasks. Advances in neural information processing systems **33**, 9459–9474 (2020)

[3] Xie, X., Liu, H., Hou, W., Huang, H.: A brief survey of vector databases. In: 2023 9th International Conference on Big Data and Information Analytics (BigDIA), pp. 364–371 (2023). IEEE

[4] Hagberg, A., Swart, P.J., Schult, D.A.: Exploring network structure, dynamics, and function using networkx. Technical report, Los Alamos National Laboratory (LANL) (2007)

[5] Chassin, D.P., Fuller, J.C., Djilali, N.: Gridlab-d: an agent-based simulation framework for smart grids. Journal of Applied Mathematics **2014**(1), 492320 (2014)

[6] Butters, O., Robson, C., Smith, B.: Openclim: A national scale framework for evaluating the effects of climate change for socio-economic scenarios and adaptation policies. In: EGU General Assembly Conference Abstracts, p. 14835 (2023)

[7] Ma, H., Zhang, Y., Sun, S., Liu, T., Shan, Y.: A comprehensive survey on nsga-ii for multi-objective optimization and applications. Artificial Intelligence Review **56**(12), 15217–15270 (2023)

[8] Leibo, J.Z., Vezhnevets, A.S., Diaz, M., Agapiou, J.P., Cunningham, W.A., Sunehag, P., Haas, J., Koster, R., Duéñez-Guzmán, E.A., Isaac, W.S., et al.: A theory of appropriateness with applications to generative artificial intelligence. arXiv preprint arXiv:2412.19010 (2024)

[9] Manivannan, A., Yow, W.Q., Bouffanais, R., Barrat, A.: Are the different layers of a social network conveying the same information? EPJ Data Science **7**(1), 1–26 (2018)

[10] Jasny, L., Waggle, J., Fisher, D.R.: An empirical examination of echo chambers in us climate policy networks. Nature Climate Change **5**(8), 782–786 (2015)

[11] Dong, Z., Lu, Z., Yang, Y.: Fine-tuning a large language model for automating computational fluid dynamics simulations. Theoretical and Applied Mechanics

Letters, 100594 (2025)

[12] Herzog, R.H., Degkwitz, T., Verma, T.: The urban model platform: A public backbone for modeling and simulation in urban digital twins. arXiv preprint arXiv:2506.10964 (2025)

[13] Gao, P., Wang, Y., Wang, H., Song, C., Ye, S., Wang, X.: A pareto front-based approach for constructing composite index of sustainability without weights: A comparative study of implementations. Ecological Indicators **155**, 110919 (2023)

[14] Giagkiozis, I., Fleming, P.J.: Pareto front estimation for decision making. Evolutionary computation **22**(4), 651–678 (2014)
